# Supplementary material for: Causal Relationship between Aging and Anorexia Nervosa: A White-Matter-Microstructure-Mediated Mendelian Randomization Analysis
Source: Biomedicines. 2024 Aug 16;12(8):1874. doi: 10.3390/biomedicines12081874 (PMC11351342; doi:10.3390/biomedicines12081874)
Supplement: Supplementary file 1 [file biomedicines-12-01874-s001.zip › Supplementary Figure/Figure S8/Leaveoneout plot of MR analysis for TL to ubm-b-1496.pdf]

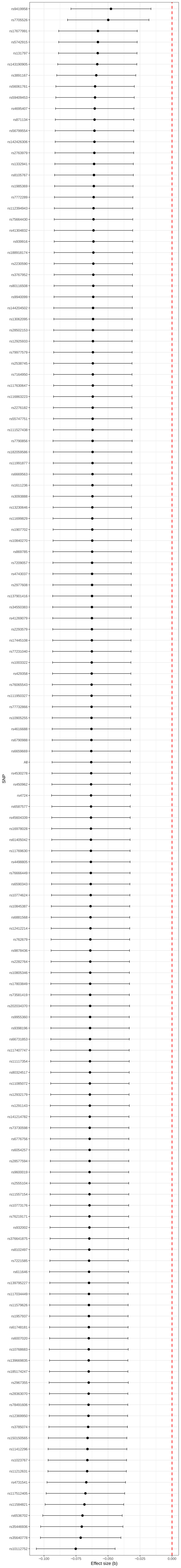
rs9419958
rs7705526
rs17677991
rs5742915
rs131797
rs143190905
rs3891167
rs56061761
rs59409453
rs4695407
rs871134
rs56799554
rs142426306
rs2763979
rs1332941
rs8105767
rs1985369
rs7772289
rs112394423
rs75664430
rs41304832
rs939916
rs188918174
rs2230590
rs3767952
rs80116508
rs9940099
rs144204502
rs13062095
rs28502153
rs12925933
rs79977579
rs2538745
rs7164950
rs117630647
rs116863223
rs2276182
rs55747751
rs111527438
rs7790856
rs182059586
rs11991877
rs6669563
rs1611236
rs3093888
rs13230646
rs11699829
rs1907702
rs10840270
rs869785
rs7209057
rs4743037
rs2977608
rs137901416
rs34550383
rs41269079
rs2293579
rs17445108
rs77231040
rs1003322
rs429358
rs76065543
rs111950327
rs77732866
rs10905255
rs4616688
rs6790988
rs6659669
All
rs4530278
rs450962
rs4724
rs6587577
rs45604339
rs16978028
rs61405042
rs11769630
rs4498805
rs76666449
rs6590343
rs10774624
rs10845387
rs6881568
rs12412214
rs762679
rs9878436
rs2282764
rs10805346
rs17803849
rs73581419
rs202034370
rs9955360
rs9398196
rs66731853
rs117407747
rs11117354
rs80324517
rs11085072
rs12932179
rs1291143
rs141214782
rs73730598
rs6776756
rs6054257
rs28577594
rs9600019
rs2555104
rs11557154
rs10773176
rs76219171
rs932002
rs376641875
rs8102497
rs7221585
rs611646
rs139795227
rs117034449
rs11579626
rs1957937
rs61748181
rs6007020
rs10768683
rs139669835
rs185174247
rs2967355
rs28363070
rs78491606
rs12369950
rs3785074
rs150150565
rs11412296
rs1023767
rs112126631
rs4731541
rs117512405
rs11584821
rs6536702
rs35446936
rs35640778
rs10112752
SNP
−0.100
−0.075
−0.050
−0.025
0.000
Effect size (b)
